# On the Exponential Inequality for Weighted Sums of a Class of Linearly Negative Quadrant Dependent Random Variables

**DOI:** 10.1155/2014/748242

**Published:** 2014-01-27

**Authors:** Guodong Xing, Shanchao Yang

**Affiliations:** School of Mathematics and Statistics, Guangxi Normal University, Guilin 541004, China

## Abstract

The exponential inequality for weighted sums of a class of linearly negative quadrant dependent random variables is established, which extends and improves the corresponding ones obtained by Ko et al. (2007) and Jabbari et al. (2009). In addition, we also give the relevant precise asymptotics.

## 1. Introduction

Lehmann [1] introduced a natural definition of negative dependence: two random variables *X* and *Y* are said to be negative quadrant dependent (NQD, say) if *P*(*X* > *x*, *Y* > *y*) ≤ *P*(*X* > *x*)*P*(*Y* > *y*) for all *x*, *y* ∈ **R**. Based on the concept of NQD, another notion of negative dependence was formulated by Newman [2] as follows: a sequence {*X*_*i*_, 1 ≤ *i* ≤ *n*} of random variables is said to be linearly negative quadrant dependent (LNQD, say) if, for any disjoint subsets *A*_1_ and *A*_2_ of {1,2,…, *n*} and positive *r*_*j*_'s, ∑_*i*∈*A*_1__ *r*_*i*_ *X*_*i*_, and ∑_*j*∈*A*_2__ *r*_*j*_ *X*_*j*_ are NQD. Recall that a finite family of random variables {*X*_*i*_, 1 ≤ *i* ≤ *n*} is said to be negatively associated (NA, say) if, for every pair of disjoint subsets *B*_1_ and *B*_2_ of {1,2,…, *n*},

(1) $$\begin{array}{c} \text{Cov}\left\{f_{1}\left(X_{i},i\in B_{1}\right),f_{2}\left(X_{j},j\in B_{2}\right)\right\}\leq 0, \end{array}$$

whenever *f*_1_ and *f*_2_ are coordinatewise increasing and the covariance exists. An infinite family is NA if every finite subfamily is NA. The concept of negative association was introduced by Joag-Dev and Proschan [3]. It is obvious to observe that NA sequences are LNQD and LNQD sequences are not necessarily NA, as it can be seen from the examples in Newman [2] or Joag-Dev and Proschan [3]. Hence, it is of interest to investigate the exponential inequality and its relevant result for LNQD sequences.

It is well-known that the exponential inequalities for partial sum ∑_*i*=1_^*n*^(*X*_*i*_ − *EX*_*i*_) play a very important role in various proofs of limit theorems. One can refer to Yang and Wang [4], T.-S. Kim and H.-C. Kim [5], Sung [6], Jabbari et al. [7], Xing et al. [8], Sung [9], and so on for further comprehension. As for the limit results about LNQD sequence, one can refer to Newman [2], Zhang [10], H. Kim and T. Kim [11], Wang and Zhang [12], and references therein.

Recently, Ko et al. [13] gave a Bernstein-Hoeffding type inequality for uniformly bounded LNQD random variables, by which they obtained the almost sure convergence rate *O*(1)*n* ^−1/2^(*p*_*n*_log⁡*n*)^1/2^ of ∑_*i*=1_^*n*^ *X*_*i*_/*n*, where *p*_*n*_ → *∞* as *n* → *∞*. Motivated by the paper above, we establish the exponential inequality for weighted sums of uniformly bounded LNQD random variables. The result obtained extends and improves the corresponding ones given by Ko et al. [13] and Jabbari et al. [7]. Furthermore, we give the precise asymptotics with respect to the rate *n* ^−1/2^(log⁡*n*)^1/2^.

Throughout this paper, we always suppose that *C* and *C*_1_ denote positive constants independent of *n* but whose value may vary over cases, [*x*] denotes the integral part of *x*, *S*_*n*_ = ∑_*i*=1_^*n*^ *X*_*i*_, *σ*_*n*_^2^ = *ES*_*n*_^2^, and *u*(*n*) = sup⁡_*i*≥1_∑_*j*:|*j*−*i*|≥*n*_|Cov(*X*_*i*_, *X*_*j*_)| and denote log⁡*n* = ln⁡(*n*∨*e*). This paper is organized as follows.  Section 2 contains our main results.  Section 3 contains the corresponding proofs.

## 2. Main Results

In this section, our main results will be given. For formulation of the theorems obtained, some assumptions are needed, which are listed below.

(A1) Let {*X*_*i*_, *i* ≥ 1} be a sequence of stationary LNQD random variables with |*X*_*i*_ | ≤*M* and let {*a*_*ni*_ : 1 ≤ *i* ≤ *n*, *n* ≥ 1} be a triangular array of numbers satisfying |*a*_*ni*_ | ≤*M*_1_, where *M* and *M*_1_ are generic positive constants.
(A2) Let {*p*_*n*_, *n* ≥ 1} be a positive integer sequence satisfying *p*_*n*_ ≤ *n*/2 and *p*_*n*_ → *∞* as *n* → *∞*.

**Theorem 1**

Suppose that the assumption (A1) holds. Then for any 0 < *ε* < 1, one has

(2) $$\begin{array}{c} P\left(\left|\sum_{i=1}^{n}a_{ni}\left(X_{i}-EX_{i}\right)\right|>n\varepsilon \right)\leq C_{1}\exp\left\{-\frac{n\varepsilon^{2}}{16C}\right\}, \end{array}$$

where *C*_1_ and *C* are positive constants.

Taking

(3) $$\begin{array}{c} \varepsilon =\varepsilon_{n}∶=4\sqrt{\frac{\left(\alpha C\log n\right)}{n}},\;\text{for}\;\text{some}\;\alpha >1, \end{array}$$

in Theorem 1, then by Borel-Cantelli lemma, we have

**Corollary 2**

Assume that the assumptions (A1) and (A2) hold. Let $p_{n}\leq \sqrt{Cn/\left(\alpha M^{2}\log n\right)}$, for some *α* > 1. Suppose that *ε* is as in (3). Then one has

(4) $$\begin{array}{c} \sum_{i=1}^{n}a_{ni}\left(X_{i}-EX_{i}\right)=O\left(1\right){\left(n\log n\right)}^{1/2}\;a.s. \end{array}$$

In particular, one has

(5) $$\begin{array}{c} \sum_{i=1}^{n}\left(X_{i}-EX_{i}\right)=O\left(1\right){\left(n\log n\right)}^{1/2}\;a.s., \end{array}$$

when *a*_*ni*_ = 1 for 1 ≤ *i* ≤ *n*.

**Remark 3**

(1) Theorem 1 generalizes Theorem 2.1 in Ko et al. [13] to weighted case. On the other hand, by (5), we can obtain that the strong convergence rate of ∑_*i*=1_^*n*^(*X*_*i*_ − *EX*_*i*_)/*n* is *O*(1)*n* ^−1/2^log⁡^1/2^ *n*, which is obviously faster than the corresponding one *n* ^−1/2^(*p*_*n*_log⁡*n*)^1/2^ that Ko et al. [13] obtained, where *p*_*n*_ ≤ *n*/2 and *p*_*n*_ → *∞* as *n* → *∞*.

(2) Since LNQD sequences are strictly weaker than NA sequences, as mentioned in Section 1, Theorem 1 extends Theorem 2.1 in Jabbari et al. [7] from strictly stationary negatively associated setting to weighted LNQD case. In addition, by the analysis mentioned above, we know that the strong convergence rate *O*(1)*n* ^−1/2^log⁡^1/2^ *n* of ∑_*i*=1_^*n*^(*X*_*i*_ − *EX*_*i*_)/*n* is much faster than the relevant one *O*(1)*n* ^−1/3^(log⁡*n*)^2/3^ Jabbari et al. [7] obtained only for the special case of geometrically decreasing covariances.

(3) For the sequence of extended negatively dependent (END, say) or wide orthant dependent (WOD, say) random variables, similar results can also be obtained.

Regarding the convergence rate *n* ^−1/2^log⁡^1/2^ *n* obtained in (5), we can give the following precise asymptotics.

**Theorem 4**

Let {*X*_*i*_, *i* ≥ 1} be a sequence of identically distributed LNQD random variables with *EX*_1_ = 0 and *E* | *X*_1_|^2+*δ*^ < *∞* for some 0 < *δ* ≤ 1, inf⁡_*n*≥1_ *σ*_*n*_^2^/*n* > 0, *u*(*n*) ≤ *Cn* ^−*θ*^ for *some θ* > 1, and

(6) $$\begin{array}{c} \sigma^{2}∶=EX_{1}^{2}+2\sum_{i=2}^{\infty }\text{ Cov }\left(X_{1},X_{i}\right)>0. \end{array}$$

Then for *β* > −1, one has

(7) $$\begin{array}{c} \underset{\epsilon ↘0}{\lim}\epsilon^{2\beta +2}\sum_{n=2}^{\infty }\frac{{\left(\log n\right)}^{\beta }}{n}P\left(\left|S_{n}\right|\geq \epsilon \sigma \sqrt{n\log n}\right)=\frac{E{\left|N\right|}^{2\beta +2}}{\beta +1}, \end{array}$$

where *N* denotes the standard normal random variable.

## 3. Proofs

Firstly, the following lemma is needed, which will be used in what follows.

**Lemma 5 (see [10])**

Let {*X*_*i*_, *i* ≥ 1} be an LNQD sequence with zero mean and finite *u*th moment. Then, for *u* > 2, there exists a positive constant *C* which only depends on *u* such that

(8) $$\begin{array}{c} E{\left|\sum_{i=1}^{n}X_{i}\right|}^{u}\leq Cn^{u/2}\underset{1\leq i\leq n}{\max}\;E{\left|X_{i}\right|}^{u}, \end{array}$$

for any *n* ≥ 1.

Next, we give some notations used later. Define *X*_*ni*_ = *X*_*i*_ for 1 ≤ *i* ≤ *n* and *X*_*ni*_ = 0 for *i* > *n*. Set *r*_*n*_ = [*n*/(2*p*_*n*_)] + 1 and then define

(9) $$\begin{array}{c} Y_{nj}=\sum_{i=2\left(j-1\right)p_{n}+1}^{2(j-1)p_{n}+p_{n}}a_{ni}\left(X_{ni}-EX_{ni}\right),\\ Z_{nj}=\sum_{i=2\left(j-1\right)p_{n}+p_{n}+1}^{2jp_{n}}a_{ni}\left(X_{ni}-EX_{ni}\right), \end{array}$$

for *j* = 1,2,…, *r*_*n*_ and

(10) $$\begin{array}{c} S_{1n}=\sum_{j=1}^{r_{n}}Y_{nj},\;\;S_{2n}=\sum_{j=1}^{r_{n}}Z_{nj}. \end{array}$$

Clearly, ∑_*i*=1_^*n*^ *a*_*ni*_(*X*_*i*_ − *EX*_*i*_) = *S*_1*n*_ + *S*_2*n*_ and *n* < 2*r*_*n*_ *p*_*n*_ ≤ 2*n*.

Now, we can obtain the following lemma.

**Lemma 6**

Let {*X*_*i*_, *i* ≥ 1} be a sequence of stationary LNQD random variables with |*X*_*i*_| ≤ *M* and let {*a*_*ni*_ : 1 ≤ *i* ≤ *n*, *n* ≥ 1} be a triangular array of numbers satisfying |*a*_*ni*_| ≤ *M*_1_, where *M* and *M*_1_ are generic positive constants. If 0 < *λp*_*n*_ *MM*_1_ ≤ 1/2 for some *λ* > 0, then on account of Definitions (9) and (10), one has

(11) $$\begin{array}{c} E\left(\exp\left(\lambda S_{1n}\right)\right)\leq \exp\left(C\lambda^{2}n\right),\\ E\left(\exp\left(\lambda S_{2n}\right)\right)\leq \exp\left(C\lambda^{2}n\right). \end{array}$$

**Proof**

Without loss of generality, assume that *a*_*ni*_ ≥ 0. Applying *EY*_*nj*_ = 0 and *λp*_*n*_ *MM*_1_ ≤ 1/2, we have by |*λY*_*nj*_| ≤ 2*λp*_*n*_ *MM*_1_ that

(12) E(exp⁡⁡(λYnj))=∑k=0∞E(λYnj)kk!=1+∑k=2∞E(λYnj)kk!≤1+E(λYnj)2∑k=2∞1k!≤1+λ2EYnj2≤exp⁡(λ2EYnj2).

Therefore, in terms of the result above and the concept of LNQD,

(13) ∏j=1rnE(exp⁡⁡(λYnj)) ≤exp⁡(λ2∑j=1rnEYnj2) ≤exp⁡(M12λ2∑j=1rn ∑i=2(j−1)pn+12(j−1)pn+pnVar⁡(Xni)) ≤exp⁡(M12λ2∑j=1rn ∑i=2(j−1)pn+12(j−1)pn+pnEX12) ≤exp⁡(Cλ2n).

Also, since *a*_*ni*_ ≥ 0 and *λ* > 0, {*λY*_*nj*_, 1 ≤ *j* ≤ *r*_*n*_} is LNQD. Therefore, from Lemma 3.1 in Ko et al. [13], we have

(14) $$\begin{array}{c} E\left(\exp\left(\lambda S_{1n}\right)\right)\leq \prod_{j=1}^{r_{n}}E\left(\exp\left(\lambda Y_{nj}\right)\right). \end{array}$$

Combining (13) and (14), we can get the desired result. Similarly, we can get the same result for *S*_2*n*_. The proof is completed.

By Lemma 6, we can easily get the following result.

**Lemma 7**

Let {*X*_*i*_, *i* ≥ 1} be a sequence of stationary LNQD random variables with |*X*_*i*_ | ≤*M* and let {*a*_*ni*_ : 1 ≤ *i* ≤ *n*, *n* ≥ 1} be a triangular array of numbers satisfying |*a*_*ni*_ | ≤*M*_1_, where *M* and *M*_1_ are generic positive constants. If 0 < *λp*_*n*_ *MM*_1_ ≤ 1/2 for some *λ* > 0, then, for any 0 < *ε* < 1 and some *C*_1_ > 0,

(15) $$\begin{array}{c} P\left(\left|S_{1n}\right|>\frac{n\varepsilon }{2}\right)\leq C_{1}\exp\left\{-\frac{n\varepsilon^{2}}{16C}\right\},\\ P\left(\left|S_{2n}\right|>\frac{n\varepsilon }{2}\right)\leq C_{1}\exp\left\{-\frac{n\varepsilon^{2}}{16C}\right\}. \end{array}$$

**Proof**

Applying Markov inequality and Lemma 6, we obtain

(16) P(|S1n|>nε2) =P(S1n>nε2)+P(−S1n>nε2) ≤P(eλS1n>eλnε/2)+P(e−λS1n>eλnε/2) ≤C1exp⁡(Cλ2n−λnε2).

Optimizing the exponent in the term of this upper-bound, we find that *λ* = *ε*/(4*C*), so that this exponent becomes equal to −*nε* ^2^/(16*C*) as desired. The proof is completed.

**Lemma 8 (see [10])**

Under the conditions of Theorem 4, one has

(17) $$\begin{array}{c} \frac{\sigma_{n}^{2}}{n}⟶\sigma^{2},\;\;\frac{S_{n}}{\sigma_{n}}⟶N\;indistribution, \end{array}$$

where *N* is a standard normal random variable.

**Lemma 9 (see [12])**

Under the conditions of Theorem 4, we have for *x* ∈ *R*

(18) $$\begin{array}{c} \underset{x}{\sup}\left|P\left(\frac{S_{n}}{\sigma_{n}}<x\right)-\Phi \left(x\right)\right|\leq Cn^{-\delta /(2+3\delta )}, \end{array}$$

where Φ(*x*) denotes the standard normal distribution function.

Based on the above lemmas, the proofs of Theorems 1 and 4 can be given as follows.

**Proof of Theorem 1**

Let *λ* > 0 which satisfies *λp*_*n*_ *MM*_1_ ≤ 1/2; then it follows from Lemma 7 that

(19) P(|Sn|>nε)≤P(|S1n|>nε2)+P(|S2n|>nε2)≤C1exp⁡{−nε216C},

which completes the proof.

**Proof of Theorem 4**

Without loss of generality, set *σ* = 1 in what follows. Since

(20) ϵ2β+2∑n=2∞(log⁡n)βnP(|Sn|≥ϵnlog⁡n) =ϵ2β+2∑n=2∞(log⁡n)βn{P(|Sn|≥ϵnlog⁡n)−P(|N|≥ϵlog⁡n)}  +ϵ2β+2∑n=2∞(log⁡n)βnP(|N|≥ϵlog⁡n),

it is sufficient to prove that

(21) $$\begin{array}{c} \underset{\epsilon ↘0}{\lim}\epsilon^{2(\beta +1)}\sum_{n=2}^{\infty }\frac{{\left(\log n\right)}^{\beta }}{n}P\left(\left|N\right|\geq \epsilon \sqrt{\log n}\right)=\frac{E{\left|N\right|}^{2(\beta +1)}}{\beta +1}, \end{array}$$

(22) lim⁡ϵ↘0ϵ2(β+1)∑n=2∞(log⁡n)βn|P(|Sn|≥ϵnlog⁡n)−P(|N|≥ϵlog⁡n)|=0,

for any *β* > −1.

To prove (21), we need only to show that

(23) $$\begin{array}{c} \underset{\epsilon ↘0}{\lim}\epsilon^{2(\beta +1)}\sum_{n=2}^{\infty }\frac{{\left(\log n\right)}^{\beta }}{n}P\left(N\geq \epsilon \sqrt{\log n}\right)=\frac{E{\left|N\right|}^{2(\beta +1)}}{2\left(\beta +1\right)}, \end{array}$$

by noting *P*(|*N* | ≥*x*) = 2*P*(*N* ≥ *x*) for any *x* > 0. It is easy to observe that

(24) lim⁡ϵ↘0ϵ2(β+1)∑n=2∞(log⁡n)βnP(N≥ϵlog⁡n) =lim⁡ϵ↘0ϵ2(β+1)∫2∞(log⁡x)βxP(N≥ϵlog⁡x)dx =lim⁡ϵ↘0∫ϵ∞2y2β+1P(N≥y)dy =E|N|2(β+1)2(β+1),

for any *β* > −1, which implies (21).

Next, we will prove (22). Let *J*(*ϵ*) = exp⁡(*M*/*ϵ* ^2^), where *M* > 4 and 0 < *ϵ* < 1/4. Obviously,

(25) ∑n=2∞(log⁡n)βn|P(|Sn|≥ϵnlog⁡n)−P(|N|≥ϵlog⁡n)| =∑n≤J(ϵ)(log⁡n)βn|P(|Sn|≥ϵnlog⁡n)−P(|N|≥ϵlog⁡n)|  +∑n>J(ϵ)(log⁡n)βn|P(|Sn|≥ϵnlog⁡n)−P(|N|≥ϵlog⁡n)| ≤∑n≤J(ϵ)(log⁡n)βn|P(|Sn|≥ϵnlog⁡n)−P(|N|≥ϵlog⁡n)|  +∑n>J(ϵ)(log⁡n)βnP(|Sn|≥ϵnlog⁡n)  +∑n>J(ϵ)(log⁡n)βnP(|N|≥ϵlog⁡n) ∶=I1+I2+I3;

thus it suffices to prove that

(26) $$\begin{array}{c} \underset{\epsilon ↘0}{\lim}\epsilon^{2(\beta +1)}I_{1}=0,\;\;\underset{\epsilon ↘0}{\lim}\epsilon^{2(\beta +1)}I_{2}=0,\\ \underset{\epsilon ↘0}{\lim}\epsilon^{2(\beta +1)}I_{3}=0, \end{array}$$

respectively. We consider firstly *I*_1_. Set $\Delta_{n}=\sup_{x}\left|P\left(|S_{n}|\geq x\sqrt{n}\right)-P\left(|N|\geq x\right)\right|$. Noticing Lemma 8, we have Δ_*n*_ → 0 as *n* → *∞*. It follows that

(27) lim⁡ϵ↘0ϵ2(β+1)I1 ≤lim⁡ϵ↘0ϵ2(β+1)∑n≤J(ϵ)(log⁡n)βnΔn =lim⁡ϵ↘0ϵ2(β+1)(log⁡J(ϵ))β+11(log⁡J(ϵ))β+1∑n≤J(ϵ)(log⁡n)βnΔn ≤lim⁡ϵ↘0Mβ+11(log⁡J(ϵ))β+1∑n≤J(ϵ)(log⁡n)βnΔn⟶0,

which implies that lim⁡_*ϵ*↘0_ *ϵ* ^2(*β*+1)^ *I*_1_ = 0. Turn to *I*_2_. By Lemma 9, it follows that

(28) P(|Sn|≥ϵnlog⁡n) ≤|P(|Snσn|≥ϵnlog⁡nσn)−P(|N|≥ϵnlog⁡nσn)|  +P(|N|≥ϵnlog⁡nσn) ≤Cn−δ/(2+3δ)+2P(N≥ϵnlog⁡nσn) ≤Cn−δ/(2+3δ)+Cn−ϵ2/2≤Cn−ϵ2/2,

for sufficiently small *ϵ*. Thus we obtain

(29) lim⁡M→∞ϵ2(β+1)I2 =lim⁡M→∞ϵ2(β+1)∑n>J(ϵ)(log⁡n)βnP(|Sn|≥ϵnlog⁡n) ≤Clim⁡M→∞ϵ2(β+1)∑n>J(ϵ)(log⁡n)βnexp⁡(−ϵ22log⁡n) ≤Clim⁡M→∞ϵ2(β+1)∫J(ϵ)∞(log⁡x)βxexp⁡(−ϵ22log⁡x)dx ≤Clim⁡M→∞ϵ2(β+1)∫M/2ϵ2∞yβexp⁡(−ϵ22y)dy ≤Clim⁡M→∞∫M/4∞tβe−tdt⟶0.

Hence, lim⁡_*ϵ*↘0_ *ϵ* ^2(*β*+1)^ *I*_2_ → 0 when *M* → *∞*. On the other hand, noting that *M* > 4 and 0 < *ϵ* < 1/4 imply $J(\epsilon )-1\geq \sqrt{J(\epsilon )}$, we can obtain

(30) lim⁡ϵ↘0ϵ2(β+1)I3 ≤lim⁡ϵ↘0ϵ2(β+1)∫J(ϵ)∞(log⁡x)βxP(|N|≥ϵlog⁡x)dx ≤lim⁡ϵ↘0ϵ2(β+1)∫J(ϵ)−1∞(log⁡x)βxP(|N|≥ϵlog⁡x)dx ≤lim⁡ϵ↘0ϵ2(β+1)∫J(ϵ)∞(log⁡x)βxP(|N|≥ϵlog⁡x)dx ≤Clim⁡ϵ↘0∫ϵM/(2ϵ2)∞y2β+1P(|N|≥y)dy ≤C∫M/2∞y2β+1P(|N|≥y)dy⟶0 as M⟶∞,

uniformly for 0 < *ϵ* < 1/4. Thus we have lim⁡_*ϵ*↘0_ *ϵ* ^2(*β*+1)^ *I*_3_ → 0, when *M* → *∞*. Combining the earlier results together yields (26). The proof is complete.
